# Dual Targeting EZH2 and Histone Deacetylases in Human Uterine Sarcoma Cells Under Both 2D and 3D Culture Conditions

**DOI:** 10.1111/jcmm.70626

**Published:** 2025-06-04

**Authors:** Mervat M. Omran, Somayeh Vafaei, Samar Alkhrait, Qiwei Yang, Ayman Al‐Hendy

**Affiliations:** ^1^ Cancer Biology Department National Cancer Institute – Cairo University Cairo Egypt; ^2^ Department of Obstetrics and Gynecology University of Chicago Chicago Illinois USA; ^3^ Department of Family Medicine Johnston Memorial Hospital, Ballad Health Abingdon Virginia USA; ^4^ Department of Medical Sciences Khalifa University Abu Dhabi UAE

**Keywords:** EZH2 inhibitors, HDAC inhibitors, uterine sarcoma

## Abstract

Uterine sarcoma is strongly associated with poor prognosis. However, its treatment options remain limited. Tazemetostat is a potent and selective EZH2 inhibitor with limited clinical application. Entinostat is one of the strong inhibitors for HDAC1 and HDAC3. This study aimed to assess the effect of dual targeting of EZH2 and HDACs on the phenotype of uterine sarcoma cells in both 2D and 3D culture systems. The uterine sarcoma cell line (MES‐SA) was treated with varying concentrations of tazemetostat and/or entinostat for 24, 48 and 72 h. For 3D culture conditions, the cells were combined with Matrigel and seeded in V‐bottom plates and incubated for 5 days. Cell proliferation, cell cycle progression and apoptosis were evaluated. Additionally, the RNA expression, IHC staining, wound healing assay, DNMT and HDAC activity measurements were conducted. Our data showed that single‐inhibitor treatment with entinostat or tazemetostat significantly increased the cytotoxicity and significantly enhanced apoptosis concomitantly. Furthermore, both inhibitors induced cell cycle arrest in 2D and 3D culture conditions. We also demonstrated that entinostat, but not tazemetostat, suppressed the wound healing in the 2D culture. The combination treatment showed a significantly superior effect compared to single‐agent treatment. Our studies demonstrate that treatment with either entinostat or tazemetostat alone showed a potent anti‐uterine sarcoma effect in 2D and 3D culture conditions. Importantly, the combination of entinostat and tazemetostat produced superior therapeutic effects, suggesting that dual targeting EZH2 and HDACs may provide a promising treatment option for this aggressive cancer.

## 1 Introduction

Uterine sarcoma is a rare gynaecologic malignancy with poor prognosis, accounting for approximately 1% of female genital malignancies and 3%–7% of uterine tumours [1, 2]. Uterine sarcoma has a poor prognosis, and existing common treatments, such as surgery, radiotherapy and chemotherapy, are not effective [3]. The 5‐year survival rate is 50%–55% for patients with early uterine sarcoma and 8%–12% for advanced cases [4, 5]. Regardless of treatment modality, many patients exhibit resistance to currently available therapies, as evidenced by high rates of both recurrence and disease progression [6]. The molecular mechanisms underlying the origin and development of uterine sarcoma are not well understood, and effective targeted treatment options remain limited.

Enhancer of zeste homologue 2 (EZH2) and histone deacetylases (HDACs) are key epigenetic regulators of cancer progression. EZH2 is the functional enzymatic component of the multiprotein histone methyltransferase complex known as polycomb repressive complex 2 (PRC2) and is responsible for mediating H3K27 trimethylation [7]. EZH2 plays an oncogenic role in several cancer types [8]. For example, heterozygous *EZH2* mutations within the catalytic SET domain have been identified in approximately 20% of cases of germinal centre B‐cell (GCB)‐like diffuse large B‐cell lymphoma (DLBCL) and follicular lymphoma (FL) [9]. These activating mutations in EZH2 result in excessive H3K27 trimethylation, leading to abnormal silencing of PRC2 targets and triggering lymphoma development [10, 11]. These findings provide the basis for EZH2 inhibition as a specific rational therapy for germinal centre–derived B‐cell NHL. Previous research indicated that EZH2 is significantly upregulated in various types of sarcoma, including Ewing sarcoma [12] and synovial sarcoma [13], where its overexpression is associated with aggressive tumour behaviour and poor prognosis [14]. These findings highlight EZH2 as a potential therapeutic target for sarcoma treatment through pharmacologic inhibition.

Tazemetostat (EPZ‐6438, E7438) is a potent and selective EZH2 inhibitor that was first approved by the US Food and Drug Administration (FDA) in 2020. This inhibitor competes with the methyl‐group donor S‐adenosyl methionine (SAM) to suppress the enzymatic activity of EZH2. Tazemetostat inhibits both wild‐type and mutant EZH2 and exhibits high selectivity over other histone methyltransferases. Tazemetostat is one of the most potent EZH2 inhibitors described to date and demonstrates favourable pharmacokinetic properties, including good oral bioavailability in animal models [15, 16].

In a previous open‐label, phase 1 trial on a non‐Hodgkin lymphoma and advanced solid tumour patients, tazemetostat was administered at doses ranging from 100 to 1600 mg orally twice daily. The 800‐mg twice daily and 1600‐mg twice daily doses were ultimately selected for the dose expansion cohort. The 800‐mg twice daily dose was chosen to proceed to phase II trials based upon evaluation of adverse effects, clinical efficacy and pharmacokinetics [17]. In other studies, the same 800 mg dosing regimen was given to epithelioid sarcoma patients until disease progression or unacceptable toxicity [18, 19].

In a different clinical trial for investigating tazemetostat in sarcomas, primarily focused on epithelioid sarcoma (ES), the drug showed an overall response rate (ORR) of 15%, meaning that 15% of patients experienced tumour shrinkage. Most of these responses were durable (lasting for at least 6 months) particularly in patients with INI1/SMARCA4‐negative tumours, a key genetic marker for ES. These data led to the FDA approval of tazemetostat for treating advanced ES patients who are not eligible for surgical resection [18].

Alterations in tumour suppressor genes or oncogenes are not always caused by genetic mutations. They may also result from transcriptional regulation through epigenetic mechanisms, including DNA methylation/demethylation and histone acetylation/deacetylation. The balance between histone acetylation and deacetylation, mediated by HATs and HDACs, respectively, is usually well regulated, but the balance is often upset in diseases such as cancer. On the other hand, in the context of sarcoma, HDACs upregulation refers to an increased expression of HDAC enzymes, which are often observed in sarcoma tumours and uLMS as we found in our previous publication [20]. This abnormal upregulation contributes to aggressive behaviour by silencing tumour suppressor genes, promoting cell proliferation and facilitating cancer progression. These findings position HDACs as potential therapeutic targets, where pharmacological inhibition of HDACs may be used to counteract these effects and suppress the tumour phenotype [21].

Conventional HDACs are composed of 11 members which require Zn^2+^as a cofactor for their deacetylase activity and are divided into four classes based on their sequence homology and function characteristics [22]. Entinostat (MS‐275, SNDX‐275) is one of the strong inhibitors for HDAC1 and HDAC3; vorinostat is used to treat cutaneous T‐cell lymphoma; tucidinostat (Chidamide, HBI‐8000, CS‐055) is a low nanomolar inhibitor of HDAC1, 2, 3 and 10.

In a previous study targeting advanced breast cancer patients, entinostat was used as monotherapy or in combination with exemestane. The standard dose of entinostat is typically 5 mg once per week administered orally [23]. Based on data from Phase I and II studies [24, 25], the most common regimen follows a 28‐day cycle, delivering approximately 20 mg of entinostat in total; this can also be given as 10 mg every 2 weeks or a schedule of 7–15 mg weekly for 3 out of 4 weeks depending on the trial design. In another clinical trial evaluating entinostat in combination with Sorafenib for treating advanced solid tumours, entinostat was administered orally once every 2 weeks, with dose escalation from 4 mg to 6 and up to 10 mg every 2 weeks [26].

The major disadvantage of two‐dimensional (2D) culture systems is that they fail to accurately represent the complex cell–cell and cell–extracellular matrix (ECM) interactions found in vivo [27]. Thus, there is a growing need for alternative models that better mimic natural cell growth and the characteristics of diseased tissue. Although various animal models have provided valuable insights into cancer pathophysiology, they present notable limitations. Simpler animal models often fail to replicate human physiology accurately, whereas studies involving more sophisticated animals, such as primates, raise ethical concerns and incur substantial costs [28]. Generally speaking, three‐dimensional (3D) culture models are superior to 2D cell cultures, as they more closely recapitulate in vivo tissue architecture while offering a more controlled, efficient and cost‐effective alternative to animal models.

This study aimed to assess the effect of single and combination treatments using HDAC and EZH2 inhibitors on human uterine sarcoma cells under both 2D and 3D culture conditions.

## 2 Material and Methods

### 2.1 Cells, Two‐Dimensional (2D) and Three‐Dimensional (3D) Models

The uterine sarcoma cell line (MES‐SA) (ATCC, Manassas, VA, USA) was cultured and maintained in McCoy's 5A medium. Human uterine leiomyosarcoma cell line (SK‐UT‐1) (ATCC) was cultured and maintained in Eagle's minimum essential medium (EMEM). Upon reaching confluence, the cells were dissociated using a solution of 0.25% trypsin and 0.1% EDTA in HBSS without calcium or magnesium (Fisher Scientific, Waltham, MA, USA). The resulting cell pellet was collected through centrifugation, and the supernatant was carefully discarded. The cell pellet was then re‐suspended in McCoy's 5A medium, ensuring any clumps were disrupted by gentle pipetting and kept on ice. Cell counting was conducted using a TC20 automated cell counter (Bio‐Rad Laboratories, Hercules, CA, USA). For 3D culture condition, approximately 10,000 cells were combined with 2 μL of Matrigel (Corning, Corning, NY, USA) mixed with 98 μL of McCoy's 5A medium yielding a final volume of 100 μL per well. The cell mixture was subsequently added to V‐bottom plates, specifically Akura 96 Spheroid Microplates (InSphero, ME, USA), followed by centrifugation. The plates were then placed in a 37°C incubator with 5% CO_2_ for 30 min. Afterward, an additional 100 μL of medium was added to each well [29]. Following a 5‐day incubation period, the spheroids were fully grown (Figure S1).

### 2.2 Assessment of the Inhibitor‐Induced Cytotoxicity in 2D Cell Culture

Cytotoxicity was assessed using the MTT assay (3‐[4,5‐dimethylthiazol‐2‐yl]‐2,5‐diphenyltetrazolium bromide) in 2D culture conditions. MES‐SA and SK‐UT‐1cells were seeded in 96 well microtitre plates at a concentration of 3 × 10^3^ cells/well. They were left to attach for 24 h before treated with drugs. The cells were treated with different concentrations (0–100 μM) of entinostat, vorinostat and tucidinostat and (0–200 μM) of tazemetostat for 24, 48 and 72 h. Based on the IC_50_ detected by the cytotoxicity assay, the cells were treated with 1.5, 3.1, 6.25 and 12.5 μM of entinostat, vorinostat and tucidinostat in combination with different concentrations (0–100 μM) of tazemetostat for 48 h. Based on the combination index calculation, the cytotoxicity of 4.5 μM tazemetostat, 6.5 μM entinostat and their combinations was retested on MES‐SA cells for 48 h. Then, MTT labelling was used according to manufacturing instructions, and absorbance (Ab) measured spectrophotometrically at 570 nm using an ELISA microplate reader. The mean values were estimated as the percentage of cell viability as follows: Ab (treated cells)/Ab (control cells) × 100. The IC_50_ value (the concentration that produces 50% inhibition of cell growth) of each drug was calculated using dose–response curve‐fitting models (Graph‐Pad Prism software, version 9).

### 2.3 Assessment of Apoptosis Using Annexin V Assay in 2D Cell Culture

The uterine sarcoma cells (MES‐SA) were plated in a tissue culture flask, allowed to attach overnight, and then treated with 4.5 μM tazemetostat, 6.5 μM entinostat and their combinations for 24 h. Flow cytometry was performed using an LSR Fortessa running DiVa software version 8.0.2 (BD Biosciences, USA), and data were analysed using FlowJov10.8.1 (BD Biosciences). Gating strategies were established based on forward and side scatter parameters to exclude debris and doublets, followed by gating on live and apoptotic cell populations using the Annexin V Alexa Fluor 488 and propidium iodide (PI) signals. The apoptosis assay was conducted using the Annexin V/PI kit (Invitrogen, Thermo Fisher, USA; Cat. No. V13242) following the manufacturer's protocol. A minimum of 100,000 events were collected per sample to ensure robust statistical analysis.

### 2.4 Cell Cycle Analysis by Flow Cytometry (FCM) in 2D Cell Culture

Cell cycle analysis for MES‐SA cells was performed using a Cell Cycle Propidium Iodide Flow Cytometry Kit (Abcam, Cat. No. ab139418, Cambridge, UK) according to the manufacturer's instructions. The measurements were conducted on LSR Fortessa (Special Order Research Product, BD Biosciences, CA, USA) equipped with DiVa software version 8.0.2. Propidium iodide was excited using a 50mw561 laser, and the signal was collected through a 585/15 bandpass filter. Cell cycle analysis was performed using the Watson model in FlowJov10.8.1.

### 2.5 Assessment of Viability and Apoptosis in 3D Spheroid Culture

Viability assessment was conducted using the CellTiter‐Glo 3D Cell Viability Assay (Promega, Germany), which is specifically designed for determining cell viability in 3D spheroids.

Similarly, RealTime‐Glo Annexin V Apoptosis and Necrosis Assay (Promega, Germany) was performed.

### 2.6 RNA Extraction, cDNA Synthesis and Quantitative Real‐Time PCR

For RNA extraction, the miRNeasy Tissue/Cells Advanced Micro Kit (Cat. No. 217684, Qiagen, Valencia, CA) was used according to the manufacturer's instructions. Reverse transcription was conducted using the RNA to cDNA EcoDry Premix (Double Primed) Kit (Cat. No. 639549, Takara Bio, USA). qRT‐PCR was performed on the CFX Connect RT‐PCR Detection System (Bio‐Rad Laboratories, Hercules, CA) using the Advanced Universal SYBR Green qPCR Mastermix (TaKaRa, Tokyo, Japan).

The PCR primers used in the experiment are listed in Table 1. The gene expression levels were normalised to GAPDH in each experiment. The relative expression of the target gene was calculated using the $2^{-\Delta \Delta C_{T}}$ method. All experiments were conducted as two independent biological replicates, each performed in triplicates.

**TABLE 1:** List of genes and primers used for qRT‐PCR.

Gene symbol	Forward primer sequence (5′–3′)	Reverse primer sequence (5′–3′)
*GAPDH*	GTCTCCTCTGACTTCAACAGCG	ACCACCCTGTTGCTGTAGCCAA
*Ki67*	GAAAGAGTGGCAACCTGCCTTC	GCACCAAGTTTTACTACATCTGCC
*PCNA*	CAAGTAATGTCGATAAAGAGGAGG	GTGTCACCGTTGAAGAGAGTGG
*Bax*	ATG TTT TCT GAC GGC AAC TTC	AGT CCA ATG TCC AGC CCA T
*Bcl‐2*	ATCGCCCTGTGGATGACTGAGT	GCCAGGAGAAATCAAACAGAGGC
*Caspase‐3*	GGAAGCGAATCAATGGACTCTGG	GCATCGACATCTGTACCAGACC
*P‐21*	AGGTGGACCTGGAGACTCTCAG	TCCTCTTGGAGAAGATCAGCCG
*CCND1*	TCTACACCGACAACTCCATCCG	TCTGGCATTTTGGAGAGGAAGTG
*CD40*	CATCCAGTCTCCCAACTTGTAT	CGGAAGGTCTGGTGGATATTAC
*PITX2*	GGCACTAAAGAAAGGGAGAGAA	CACTGAGGACATCCCTTTGAA

### 2.7 Formation of Paraffin‐Embedded Spheroid Blocks

MES‐SA spheroids were sent to the Organoid and Primary Culture Research Core at the University of Chicago. Paraffin‐embedded spheroid blocks were prepared following techniques developed in the Organoid Core. Briefly, MES‐SA spheroids were collected in a microcentrifuge tube and fixed in 10% paraformaldehyde for 60 min. Then 1% agarose solution (Agarose I, VWR Corporation, USA) was added, mixed gently with spheroids and allowed to solidify for about 1 h at room temperature. The agarose gels with spheroids were sliced longitudinally and processed on a tissue processor (Leica ASP6025, Germany) prior to embedding in paraffin. Sections were cut at 5 μm and stained with haematoxylin and eosin (H&E). Immunohistochemical (IHC) staining was performed at the University of Chicago Pathology Core Facility. The proliferation marker, including Ki67, was investigated to assess cellular proliferation within the spheroids. Antibody information is summarised in Table 2. The expression levels of Caspase 3 and Bax were also analysed to determine the apoptotic activity. The slides were scanned and analysed using the Aperio ImageScope colocalization algorithm—Pathology Slide Viewing Software.

**TABLE 2:** List of primary antibodies used in the experiments.

Antibodies names	Type	Cat#	Using in study	Optimised dilution	Source
Anti‐Ki67 antibody [SP6]	Primary	ab16667	IHC	1:200	Abcam, MA, USA
Anti‐caspase‐3 antibody [E87]	Primary	ab32351	IHC	1:100	Abcam, MA, USA
Anti‐Bax antibody [E63]	Primary	ab32503	IHC	1:800	Abcam, MA, USA

### 2.8 Wound Healing Migration Assay

MES‐SA cells were detached using a non‐enzymatic cell dissociation buffer, centrifuged and the supernatant was aspirated. The cells were re‐suspended in serum‐free cell culture media containing 0.1% bovine serum albumin (BSA). A total of 100 μL of the cell solution (2 × 10^6^ cells/ml) was plated in each well of a 6‐well plate and incubated for 24 h at 37°C and 5% CO_2_ to allow cell attachment. Pipette tips were used to make a fine scratch in each well. Positive and negative controls were prepared, and the other wells were treated with 4.5 μM tazemetostat, 6.5 μM entinostat or their combinations. After incubation for 48 h, the media was removed, and the wells were washed with saline. One millilitre of 70% ethanol was added into each well for 30 min to allow cell fixation. Ethanol was removed, and the wells were allowed to dry for 10–15 min. The wells were washed with distilled water. After drying, the wells were examined under an inverted microscope, and images were acquired.

### 2.9 Measurement of DNMT Activity

The cells and spheroids were subjected to nuclear extraction using the EpiQuik Nuclear Extraction kit (Cat. No. OP‐0002, Epigentek, NY, USA), according to the manufacturing instruction manual. The protein concentration of the nuclear extract was measured using the Protein Assay Dye Reagent Concentrate Kit (Cat. No. #5000006, Bio‐Rad Laboratories). The DNMT activity was measured using the Colorimetric ELISA Easy Kit (Cat. No. P‐3139‐96, Epigentek, NY, USA).

### 2.10 HDAC Activity Measurement

The HDAC activity was measured using the Colorimetric ELISA Easy Kit (Cat. No. P‐3139‐96, Epigentek).

### 2.11 Statistical Analysis

All raw data were collated in a Microsoft Excel database, while Prism 9 was used for statistical analysis. The data results are presented as mean ± SD or mean ± SEM (standard error of the mean). The statistical significance of the differences was determined using a *t*‐test or one‐way/two‐way analysis of variance (ANOVA) followed by Tukey comparisons. Statistical significance was defined as *p*‐values < 0.05, which were considered significant in a two‐tailed analysis.

## 3 Results

### 3.1 Cytotoxicity Screening With Single‐Agent Treatments

Based on our literature review, we selected three different HDAC inhibitors (vorinostat, tucidinostat and entinostat) and one EZH2 inhibitor (tazemetostat) in our study. We conducted cytotoxicity screening of these compounds in MES‐SA 2D cultures at 24, 48 and 72 h. Vorinostat increased cell toxicity with an IC_50_ value of 6.5 μM after 48 and 72 h treatments (Figure S2A). Tucidinostat showed IC_50_ values of 7.2, 6 and 6.26 μM for 24, 48 and 72 h, respectively (Figure S2B). While entinostat exhibited a cytotoxic effect with IC_50_ values of 50, 12.5 and 6.25 μM after 24‐, 48‐ and 72‐h treatment, respectively (Figure S2C). On the other hand, tazemetostat induced toxicity with IC_50_ values of 115, 113 and 80.5 μM at the three different treatment times, respectively (Figure S2D).

To extend our study to a different uterine cell line, SK‐UT‐1 cells were treated with various concentrations (0–100 μM) of entinostat and (0–100 μM) of tazemetostat for 24, 48 and 72 h. Tazemetostat showed IC_50_ values of 24, 18 and 17 μM for 24, 48 and 72 h, respectively (Figure S3A). Entinostat exhibited a cytotoxic effect with IC_50_ values of 20, 5 and 5 μM after 24‐, 48‐ and 72‐h treatment, respectively (Figure S3B).

### 3.2 The Effect of Combination Treatments on Cytotoxicity in MES‐SA 2D Culture

Tazemetostat was tested at concentrations ranging from 0 to 100 μM in combination with four different concentrations (1.5, 3.1, 6.25 and 12.5 μM) of each of the three HDAC inhibitors for 48 h on the MES‐SA cell line. Combination treatment of vorinostat and tucidinostat did not exert any superior effect compared to the individual compounds alone (Figure S2E,F). In contrast, the combination of tazemetostat and entinostat resulted in a marked inhibition in the cellular proliferation of the MES‐SA cell line in a concentration‐dependent manner (Figure S2G). Notably, the first significant effect of entinostat was observed at a concentration of 12.5 μM when used alone (Figure S1C). The combination of 6.25 μM entinostat with tazemetostat resulted in a decrease in cell viability at an IC_50_ value of 4.5 μM compared to the IC_50_ value of tazemetostat (113 μM) or entinostat (12.5 μM) alone. Thus, a lower dose of each in combination achieved a comparable IC_50_.

### 3.3 The Combined Treatment Regimen of Tazemetostat and Entinostat Was Synergistic in MES‐SA Cells

An evaluation of the drug interaction was carried out and the combination index (CI) was calculated, where CI = 1 indicates an additive effect, CI > 1 indicates antagonism, CI < 1 indicates synergism. A synergistic drug interaction of 4.5 μM tazemetostat and 6.25 μM entinostat was detected in the MES‐SA cell line with a combination index, CI = 0.539.

With the equation:

$$\mathrm{CI}=\left(D\right)1\;\left(D\;x\right)1+\left(D\right)2\;\left(D\;x\right)2$$

where (D*x*)1 represents the dose of the drug D1 alone that inhibits the growth of cells by *x*% and (D*x*)2 is the dose of the drug D2 alone that inhibits the growth of cells by *x*%.

### 3.4 Effect of Combination Treatment With Tazemetostat and Entinostat on Proliferation in 2D and 3D Culture Conditions

**FIGURE 1:**
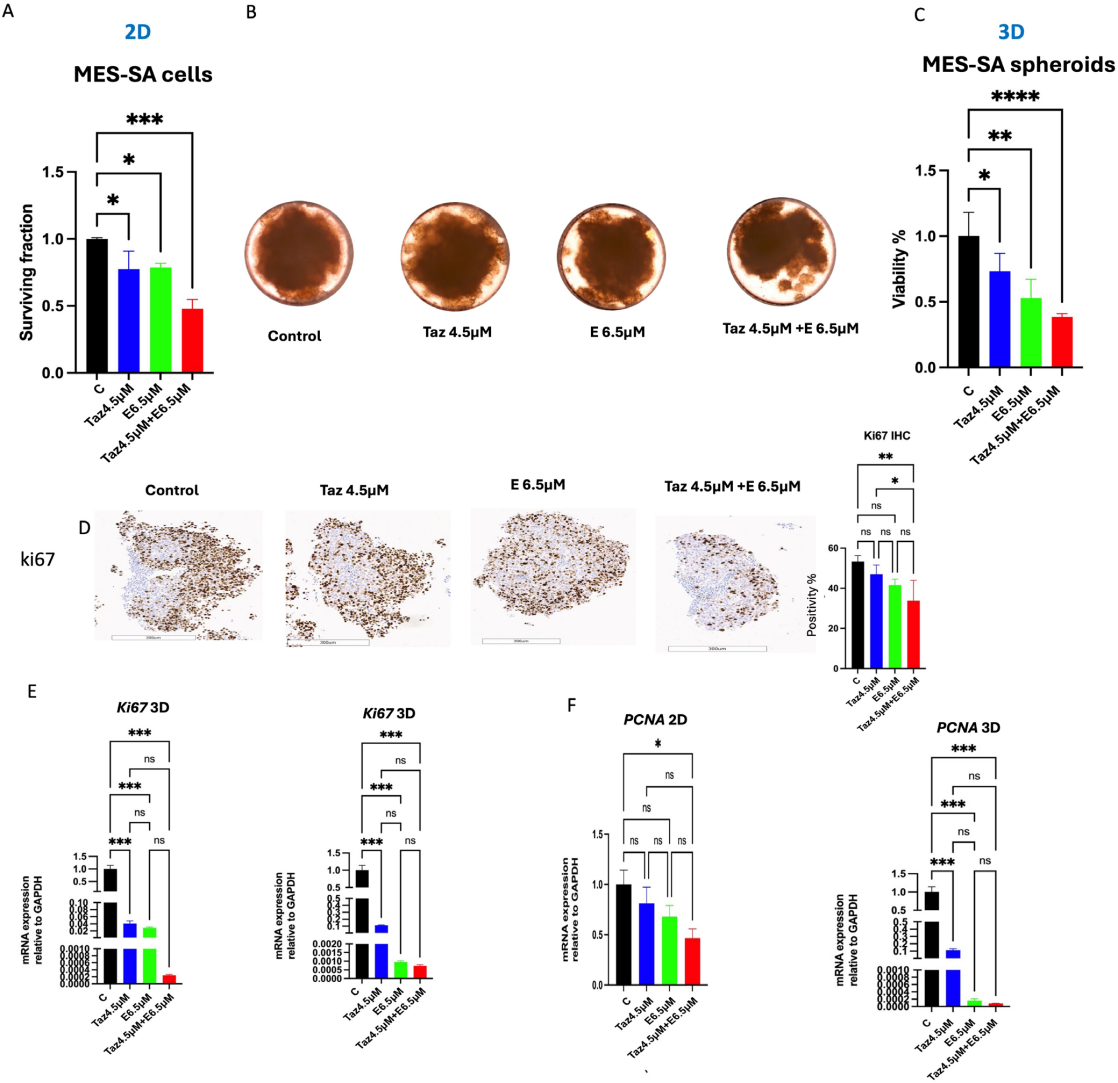
Effects of tazemetostat, entinostat and their combinations on proliferation of uterine sarcoma cells (MES‐SA) under 2D and 3D cell culture conditions after 48‐h treatment. (A) Surviving fraction of Mes‐SA cells treated tazemetostat, entinostat and their combinations. (B) Representative imaging of 3D uterine sarcoma spheroid under control and following treatments with tazemetostat (4.5 μM), entinostat (6.5 μM) and their combination. (C) Viability % of 3D Uterine sarcoma spheroids treated with tazemetostat, entinostat and their combinations. (D) Histology and immunohistochemical staining of 3D Uterine sarcoma spheroid with Ki67. The slides were scanned and analysed using the Aperio ImageScope colocalization algorithm—Pathology Slide Viewing Software. (E, F) mRNA expression of proliferation markers Ki67 (E) and PCNA (F) in 2D and 3D culture systems following treatments. Values are the means ± SD of three independent experiments performed in triplicate. Statistical significance was determined using one‐way ANOVA followed by Tukey's multiple comparison test. *p < 0.05, **p < 0.01, ***p < 0.001. C, control; E, entinostat; Taz, tazemetostat.

The surviving fraction of MES‐SA cells was decreased significantly upon exposure to 4.5 μM tazemetostat, 6.5 μM entinostat and combination treatment (24%, 21% and 50%), respectively (*p* > 0.05, 0.05 and 0.001) relative to control (Figure 1A). To confirm our finding, we repeat the study on MES‐SA 3D spheroid as described in Section 2. We noticed a decrease in size and volume of the spheroids when treated with our drug of interest and especially their combination (Figure 1B). The measuring of viability fraction of the 3D spheroids showed (25%, 50% and 60% inhibition) after treatment with 4.5 μM tazemetostat, 6.5 μM entinostat and their combination, respectively (*p* < 0.05, 0.01 and 0.001) compared to the control (Figure 1C). To confirm the finding, mRNA expression of proliferative marker genes, including *PCNA* and *Ki67*, was examined. The expression levels of *PCNA* and *Ki67* showed a significant decrease in treatment groups, especially the combination group relative to control in both 2D and 3D culture conditions (*p* < 0.001) (Figure 1E,F). At the protein level, we conducted IHC for the 3D model; Ki67 showed a significant decrease in the combination group compared to control and tazemetostat group (*p* < 0.01 and 0.05), respectively (Figure 1D).

### 3.5 The Effect of Combination Treatment With Tazemetostat and Entinostat on Apoptosis in 2D and 3D Culture Conditions

**FIGURE 2:**
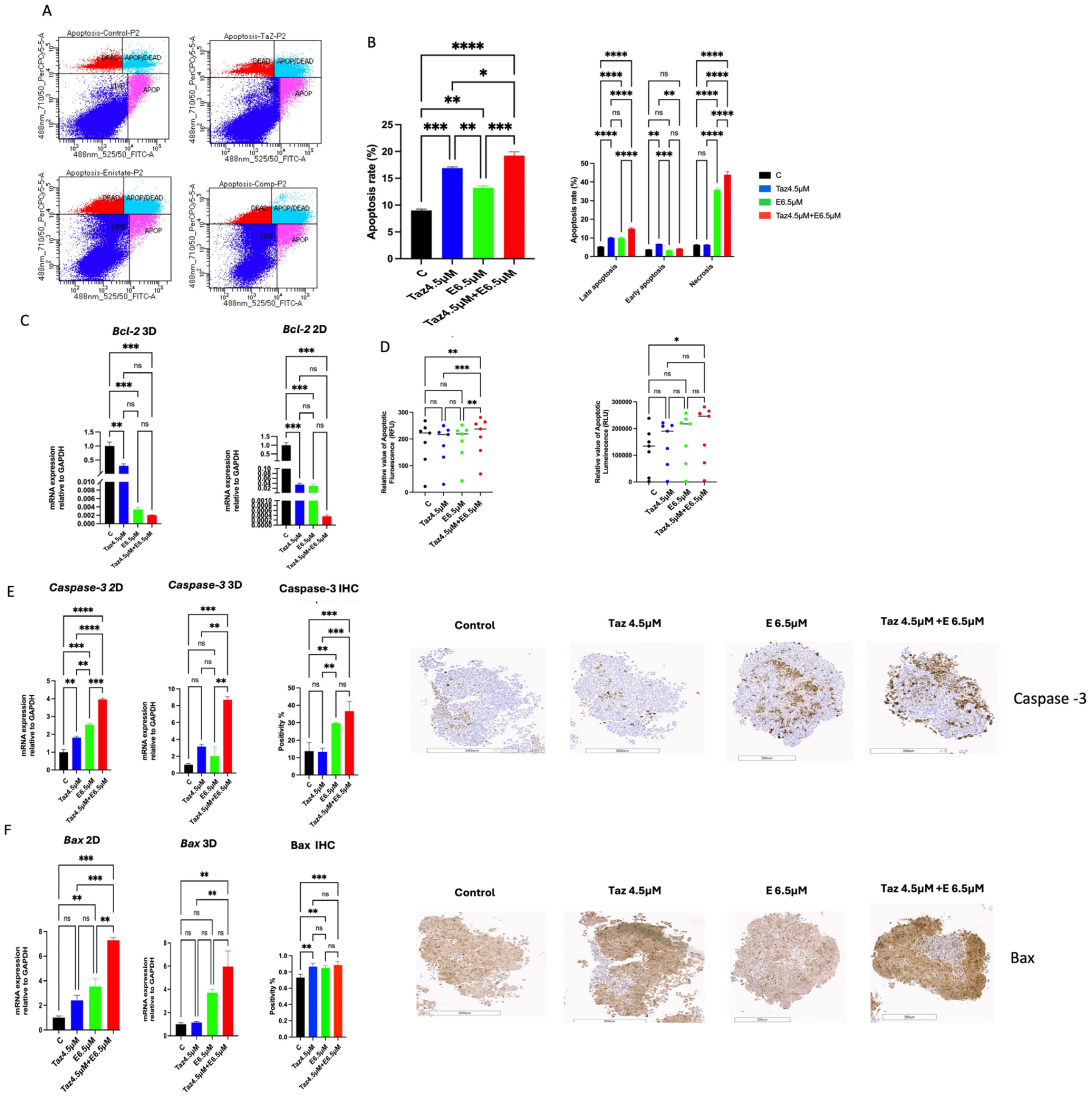
Effect of tazemetostat, entinostat and their combinations on apoptosis in uterine sarcoma cells (MES‐SA). (A) Flow cytometry scatterplots using Annexin V staining for control, 4.5 tazemetostat μM, 6.5 μM entinostat, their combinations. (B) Quantitative analysis of the total apoptosis rate and quantitative analysis of late apoptosis, early apoptosis and necrosis rate. (C) Effect of tazemetostat 4.5 μM, 6.5 μM entinostat and their combination on mRNA expression of apoptotic marker Bcl‐2 in both 2D and 3D culture conditions. (D) Relative apoptotic luminescence and florescence of 3D spheroids after exposure to 4.5 μM tazemetostat, 6.5 μM entinostat and their combination for 48 h. (E) Effect of 4.5 μM tazemetostat, 6.5 μM entinostat and their combination on mRNA expression in both 2D and 3D culture conditions and immunohistochemical staining of 3D Uterine sarcoma spheroid of Caspase‐3. (F) Effect of 4.5 μM tazemetostat, 6.5 μM entinostat and their combination treatments on mRNA expression of *BAX* in both 2D and 3D culture systems and immunohistochemical staining of BAX in 3D uterine sarcoma spheroids. The slides were scanned and analysed using the Aperio ImageScope colocalization algorithm (Pathology Slide Viewing Software). Results are expressed as means ± SD of two independent experiments performed in duplicates. Statistical significance was determined using one‐way ANOVA followed by Tukey's multiple comparison test *p < 0.05, **p < 0.01, ***p < 0.001, ****p

In the 2D culture, flow cytometry was performed using Annexin V in the control group and three treated regimens (4.5 μM tazemetostat, 6.5 μM entinostat or their combinations), as shown in Figure 2A. The single entinostat treatment resulted in a significant increase in total apoptosis and necrosis rates of 13% and 36%, respectively, compared to the control group as depicted in Figure 2B. Tazemetostat treatment alone depicted a significant increment in total apoptosis rate to almost 17% compared to the control group. Furthermore, tazemetostat combined with entinostat exhibited a more pronounced significant augmentation in total apoptosis and necrosis rates of nearly 19% and 45%, respectively, compared to the control group.

<
    0.0001 C, control; E, entinostat; Taz, tazemetostat.

Under the 3D culture condition, we measured the relative number of apoptotic luminescence and fluorescence units. Our data revealed a significant increase in apoptosis in the group treated with the combination regimen (*p* < 0.01 and 0.05, respectively) (Figure 2D). To confirm the finding, the mRNA expression of the apoptotic marker was examined. A significant decrease in the expression of *Bcl‐2* (*p* < 0.001) was observed in the treatment groups compared to the control group, as shown in Figure 2C. In addition, single and combination treatments significantly increased *Caspase‐3* and *Bax* expression compared to the control group. Moreover, the combination treatment significantly enhanced the expression of *Caspase‐3* and *Bax* compared to the single treatments in both 2D and 3D culture conditions (*p* < 0.001) (Figure 2E).

We also conducted IHC to measure the caspase‐3 protein levels in 3D spheroid cultures. Treatment with entinostat alone or in combination with tazemetostat significantly increased the levels of caspase‐3 by 20% and 25%, respectively, compared to the control (*p* < 0.001 and 0.01). In contrast, treatment with tazemetostat alone did not show a significant increase in caspase‐3 expression compared with the control group (Figure 2E). Moreover, tazemetostat, entinostat and their combination treatments significantly increased the expression of *Bax* compared to the control group (*p* < 0.01, *p* < 0.01 and *p* < 0.001) (Figure 2H).

### 3.6 The Effect of Combination Treatment With Tazemetostat and Entinostat on Cell Cycle Progress in 2D and 3D Culture Conditions

**FIGURE 3:**
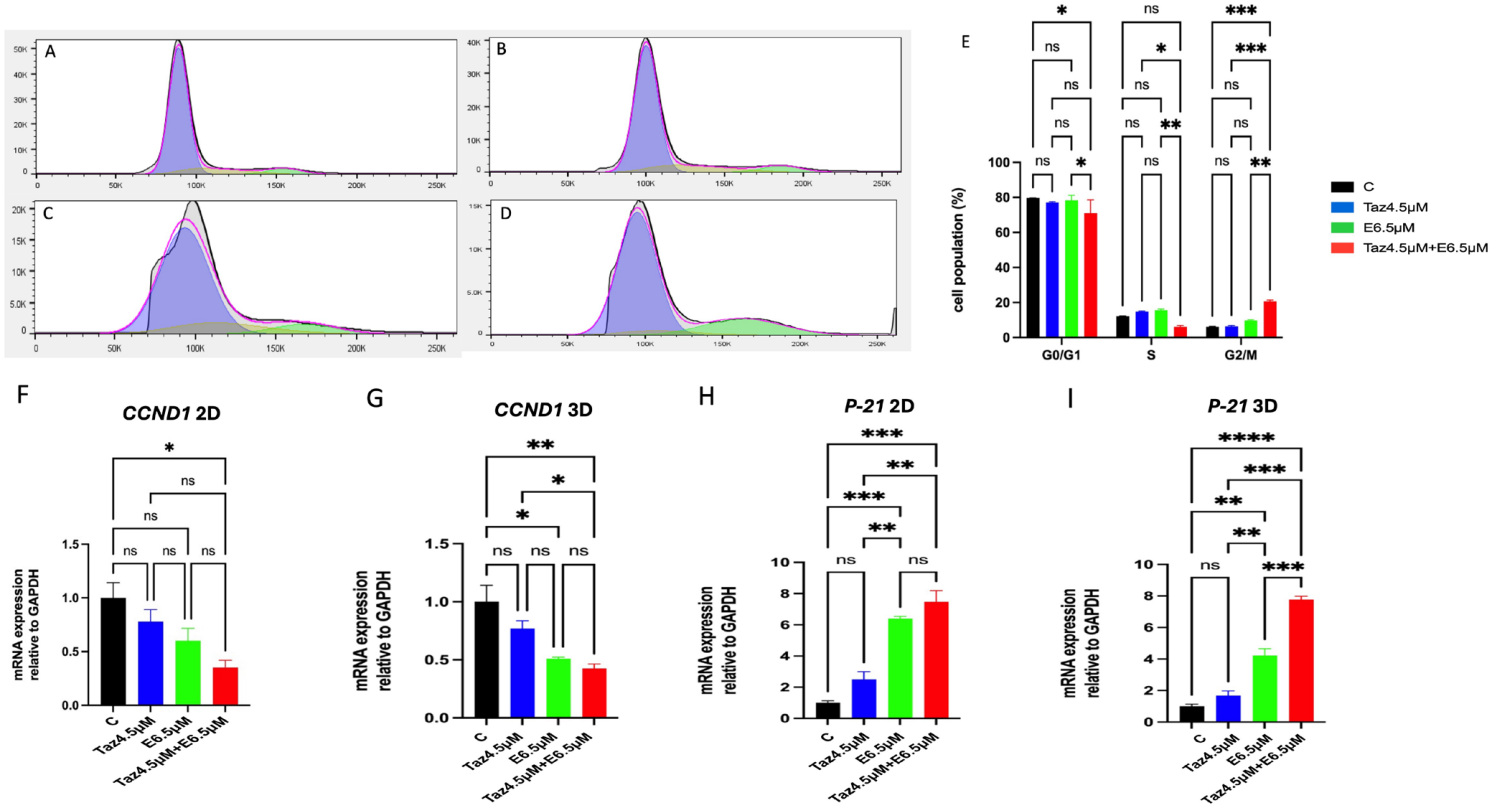
Cell cycle analysis of MES‐SA cells. (A–D) Flow cytometry histograms showing DNA content in control cells (A), cells treated with tazemetostat 4.5 μM (B), entinostat 6.5 μM (C) and their combination (D). (E) Bar charts representing the percentage of cell population in G0–G1, S and G2‐M phases in untreated cells and after treatment with tazemetostat, entinostat and their combination. Effect of tazemetostat 4.5 μM, entinostat 6.5 μM and their combination on mRNA expression of cell cycle marker. (F, G) Cyclin D1 (*CCND1*) and (H, I) *P21* in both 2D and 3D cell culture conditions. Results are expressed as means ± SD of two independent experiments performed in triplicate. Statistical significance was determined using two‐way ANOVA followed by Bonferroni test and one‐way ANOVA using Tukey's multiple comparison test. *p < 0.05, **p < 0.01, ***p < 0.001, **** *p* < 0.0001. C, control; E, entinostat; Taz, tazemetostat.

Flow cytometric analysis confirmed a significant cell cycle arrest following treatment with a combination of both 4.5 μM tazemetostat and 6.5 μM entinostat, consistent with the cell count results (Figure 3A–D). The combination regimens significantly reduced the S‐phase population, accompanied by an accumulation of cells in the G1 phase. Notably, the combination regimen resulted in a 41% inhibition in S phase fraction compared to the control group.

To confirm the finding, mRNA expression of cell cycle‐related markers was conducted. Significant decrease (50% inhibition) in the expression of CyclinD1 (*CCND1*) (*p* < 0.05 and 0.01) in the combination treated group in both 2D and 3D culture conditions, respectively, compared to the control group as shown in Figure 3F,G. Additionally, a significant increase in *P21* expression was observed in MES‐SA cells treated with both tazemetostat 4.5 μM and entinostat 6.5 μM alone or in combination. Furthermore, the combination showed a sixfold increase in *P21* expression relative to the control group under both 2D and 3D culture conditions (*p* < 0.001) (Figure 3H,I).

### 3.7 Effect of Combination Treatment With Tazemetostat and Entinostat on Inhibition of Cell Migration

**FIGURE 4:**
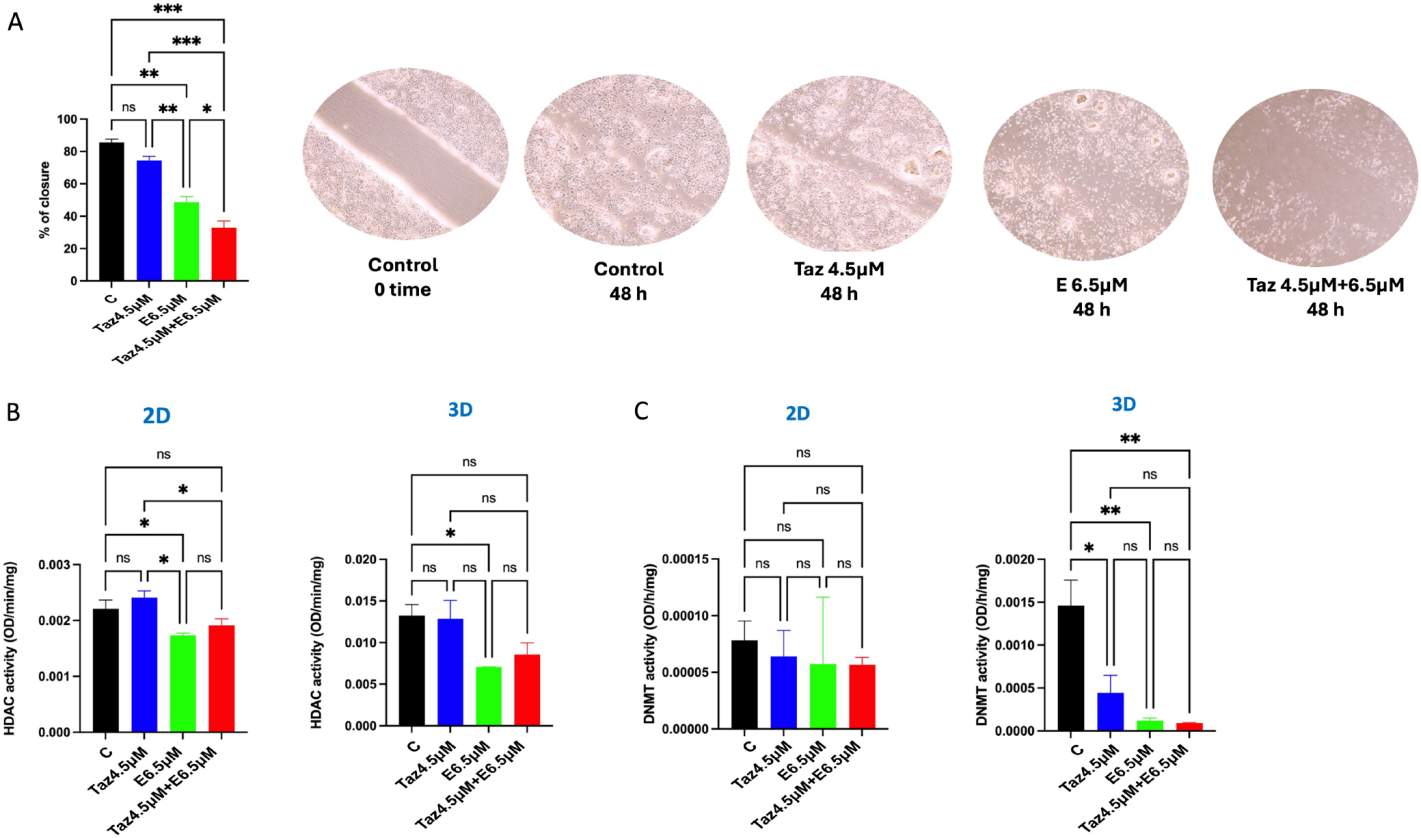
The effects of single and combination treatments of tazemetostat and entinostat on wound healing and epigenetic enzyme activity in uterine sarcoma cells. (A) Wound healing assay for uterine sarcoma cells (MES‐SA) treated with 4.5 μM tazemetostat, 6.5 μM entinostat and their combination for 48 h. (B) Total HDAC activity of MES‐SA cells treated with 4.5 μM tazemetostat, 6.5 μM entinostat and their combination in both 2D and 3D cell culture systems. (C) Total DNMT activity of MES‐SA cells treated with 4.5 μM tazemetostat, 6.5 μM entinostat and their combination under both 2D and 3D cell culture conditions. The bar graphs represent the mean ± SEM of three technical replicate measurements of three independent biological replicates. *p < 0.05, **p < 0.01, ***p < 0.001. C, control; E, entinostat; ns, no significant difference; Taz, tazemetostat.

We conducted a wound healing assay, and our data demonstrated that both 6.5 μM entinostat and their combination with 4.5 μM tazemetostat exhibited a significant antiangiogenic effect relative to the control (*p* < 0.01, 0.001) respectively, with qualitatively higher activity noticed in wells treated with the combination (Figure 4A).

### 3.8 Effect of Combination Treatment With Tazemetostat and Entinostat on DNMT and HDAC Activities

By measuring the total HDAC activity of cells treated with 4.5 μM tazemetostat, 6.5 μM entinostat and their combination, we observed a significant inhibition of HDAC activity in the group treated with 6.5 μM entinostat, especially in the 3D culturing condition, relative to the control group (*p* < 0.05) (Figure 4B). In addition, by measuring the total DNMT activity in the group treated with tazemetostat 4.5 μM, entinostat 6.5 μM and their combination, we detected a significant inhibitory effect on DNMT activity in MES‐SA cells treated with either of the two single drugs and their combination in the 3D culturing model (*p* < 0.01) (Figure 4C).

To assess whether tazemetostat and entinostat effectively inhibit EZH2 and HDACs, respectively, we performed real‐time PCR for *PITX2* and *CD40*, known targets of EZH2 [30] and HDACs [31, 32] respectively. A significant decrease in *PITX2* expression was observed in the tazemetostat‐treated and combination groups compared to the control in both 2D and 3D cultures (*p* < 0.05 and *p* < 0.01, respectively), as shown in Figure S3A,B. Additionally, the entinostat‐treated and combination groups showed a significant increase in *CD40* expression compared to the control in both 2D and 3D cultures (*p* < 0.01 for both), as shown in Figure S3C,D.

## 4 Discussion

Entinostat (MS‐275), a synthetic benzamide derivative of HDACI with oral bioavailability, potently and selectively inhibits class I and IV HDAC enzymes. The pharmacokinetics of entinostat are linear across dosages ranging from 2 to 12 mg/m^2^, and it possesses a prolonged half‐life ranging from 33 to 150 h [33]. Preclinical studies, both in vitro and in vivo, have demonstrated promising anti‐tumour activity of entinostat in various cancers, including lung, prostate, breast and pancreatic cancer [34]. In 2013, the combination of entinostat and letrozole was shown to restore the responsiveness of aromatase inhibitors in letrozole‐resistant MCF‐7Ca xenografts through modulating Her‐2, with outperforming treatment with either agent alone [35]. Moreover, entinostat has been found to enhance the anti‐tumour efficacy in combination with HER2‐targeted agents, such as lapatinib and trastuzumab in patients with HER2‐overexpressing breast cancer cells that are resistant to trastuzumab‐based treatment [36].

Despite the promising data, entinostat has not yet been approved for clinical use by any regulatory agency [25]. However, it has granted ‘breakthrough designation’ status from the US FDA when used in combination with exemestane for the treatment of advanced breast cancer [25]. Ongoing research includes a number of Phase I and II combination trials investigating the use of entinostat in combination with other drugs that modify epigenetic mechanisms. For example, in 2013, a randomised, double‐blind, placebo‐controlled phase II study evaluated entinostat combined with the aromatase inhibitor exemestane versus exemestane alone (ENCORE301 trial, NCT00676663). The results showed that the combination was generally well‐tolerated in patients with ER+ advanced breast cancer and suggested that acetylation changes may provide an opportunity to maximise clinical benefit with entinostat [37].

A previous study evaluated entinostat against three rhabdomyosarcoma cell lines using a 96‐h drug exposure protocol. Entinostat alone or in binary combination with vincristine, actinomycin D or cyclophosphamide was tested in one alveolar rhabdomyosarcoma (ARMS) and two embryonal rhabdomyosarcoma (ERMS) xenograft models. The treatment by entinostat showed modest antitumour activity in only one of four models at a dose and schedule relevant to human exposure. The conclusion was that adding entinostat to standard cytotoxic agents did not enhance efficiency compared to the cytotoxic agents alone [38]. A more recent Phase II study evaluating the efficacy and safety of entinostat in patients with relapsed or refractory abdominal neuroendocrine tumours demonstrated that weekly administration of 5 mg entinostat led to prolonged stable disease and reduced the tumour growth rates by 32%–80%, with an acceptable safety profile (ClinicalTrials.gov Identifier: NCT0311988) [39].

Hence, adding epigenetic therapy may be an effective approach to targeting resistance pathways in uterine sarcoma. Our research team is the first to introduce a combination of EZH2 inhibitors and histone deacetylase inhibitors as a potential epigenetic therapy for uterine sarcoma [40]. The current study shows that both 6.5 μM entinostat and its combination with 4.5 μM tazemetostat exhibit significant cytotoxicity in the uterine sarcoma cell line by inducing cell cycle arrest and promoting apoptosis. This finding aligns with previous studies testing entinostat on Ewing sarcoma tumours [41]. Additionally, recent research suggests that entinostat enhances the efficacy of chemotherapy, particularly cisplatin in small cell lung cancer by inducing S‐phase arrest and increasing apoptosis [42]. Our study also demonstrates that entinostat exhibits antimigration effects detected by a wound healing assay at a concentration of 6.5 μM, consistent with findings by Sultana et al. [43], where entinostat at concentrations of 0.01, 0.1 and 10 μM significantly suppressed cellular migration in an odontoblast‐like cell line.

The second agent we employed in this study is tazemetostat, which is a first‐in‐class US Food and Drug Administration (FDA)‐approved oral EZH2 inhibitor for FL and epithelioid sarcoma (ES) [44]. In July 2019, the FDA granted priority review to tazemetostat for the treatment of patients with metastatic or locally advanced epithelioid sarcoma who were not eligible for complete resection. On January 23, 2020, the FDA granted accelerated approval to tazemetostat for this use. Tazemetostat is the first treatment option specifically approved by the FDA for this rare subtype of soft tissue sarcoma, which often occurs in young adults [45]. Some previous studies mention that undifferentiated uterine sarcoma (malignant rhabdoid tumour of the uterus) may be responsive to the EZH2 inhibitor tazemetostat [46, 47].

As an oral medication, tazemetostat has a 33% bioavailability. It is hepatically metabolised by CYP3A to form its two major inactive metabolites M5 (EPZ‐6930) and M3 (EPZ006931), with M5 being further metabolised by CYP3A. This metabolic pathway accounts for the drug–drug interactions seen with tazemetostat. The mean terminal half‐life of tazemetostat is 3.1 h, and it is excreted primarily through faeces (79%) and urine 15% [44].

This study demonstrates that tazemetostat exerts a cytotoxic effect on uterine sarcoma cell lines with IC_50_ values of 115, 113 and 80.5 μM after exposure for 24, 48 and 72 h as a single agent, respectively. When combined with entinostat, the IC_50_ value was reduced to 4.5 μM after 48 h in the MES‐SA cell line. In the SK‐UT‐1 cell line, tazemetostat exhibited IC_50_ values of 24, 18 and 17 μM at 24, 48 and 72 h, respectively. Meanwhile, entinostat showed cytotoxic effects with IC_50_ values of 20, 5 and 5 μM following 24, 48 and 72 h of treatment, respectively. This finding is consistent with a previous study showing that treatment of synovial sarcoma (Fuji or HS‐SY‐II) cell lines with tazemetostat led to a concentration‐dependent decrease in proliferation over a 14‐day assay (IC_50_ values on Day 14 of 0.15 μmol/L [95% CI: 0.065–0.35] and 0.52 μmol/L [95% CI: 0.36–0.75], respectively) [16].

Furthermore, our study suggests that the combination of tazemetostat and entinostat induces cell cycle arrest by inhibiting Cyclin D1 expression and stimulating P21 expression. It also promotes apoptosis via downregulation of Bcl‐2 and upregulation of Bax and caspase 3, affecting both intrinsic and extrinsic apoptotic pathways. Similarly, tazemetostat has been shown to induce cell cycle arrest and apoptosis in lymphoma cells in preclinical models [48]. Additionally, tazemetostat has been found to enhance 5‐FU‐induced apoptosis by upregulating PUMA (p53 upregulated modulator of apoptosis) and activating the mitochondrial apoptosis pathway in colorectal cancer [49].

A recent study found that tazemetostat induced antitumorigenic effects, including increased apoptosis and reduced wound healing capacity in renal cell carcinoma [50]. On the other hand, the current study found that tazemetostat alone had an insignificant effect on wound healing, whereas its combination with entinostat resulted in a significant reduction in wound healing capacity.

To assess whether tazemetostat and entinostat effectively inhibit EZH2 and HDACs, respectively, we examined the expression of their downstream targets. A significant decrease in *PITX2* expression, a target of EZH2, was observed in the tazemetostat‐treated and combination groups compared to the control in both 2D and 3D cultures (*p* < 0.05 and *p* < 0.01, respectively). Additionally, the entinostat‐treated and combination groups showed a significant increase in *CD40* expression, a known HDAC target, compared to the control in both 2D and 3D cultures (*p* < 0.01 for both). Previous studies have also reported that some HDAC inhibitors increase CD40 expression [31]. Moreover, EZH2 overexpression has been shown to significantly upregulate several Wnt ligands such as PITX2, which promotes nuclear translocation of β‐catenin and PCNA, ultimately leading to increased cell proliferation [30].

Moreover, previous studies have noticed that EZH2 inhibitors enhance the viral mimicry effects of DNMT inhibition through a mechanism of NFAT: AP‐1 signalling [51].

Although the 2D and 3D results in our study led to similar conclusions, the 3D model results showed superior efficiency of the combination therapy in most markers. The 3D model is more representative of the real environmental conditions of the in vivo tumour, especially in the context of oxidative stress. Thus, our 3D model serves as a valuable tool for mimicking the physiological conditions of tumours in vivo. In comparison to 2D cell cultures, 3D cell cultures generally exhibit significantly lower drug uptake, absorption and penetration due to the complex, multi‐layered structure and presence of an extracellular matrix, creating physical barriers that hinder drug diffusion to the inner cell layers, leading to reduced drug uptake [52]. In addition, 3D cultures can exhibit significant cell‐to‐cell variability in drug exposure due to gradients in nutrient and oxygen availability within the tissue‐like structure. Additionally, the size, charge and hydrophobicity of a drug molecule can significantly impact its ability to penetrate through the 3D tissue matrix [53]. Moreover, the type and density of the extracellular matrix used in 3D cultures can influence drug diffusion and cell behaviour. For example, larger spheroids tend to have greater drug resistance due to extended diffusion distances within the tissue mass. Together, these features can create barriers to drug diffusion, leading to increased drug resistance and a more accurate representation of in vivo tissue behaviour, particularly in studies investigating drug resistance mechanisms [54].

Our 3D culture system offers several advantages, including its potential to reduce animal testing significantly, its applicability for functional assays and drug testing within a short time frame, and its ability to provide materials for cytopathology and molecular analysis. This system also allows for correlating structure with function, spatial distributions or theoretical analysis. Spheroid integrity can be easily visualised using (rapid) phase‐contrast imaging, and the protocols are highly reproducible. Furthermore, drug efficacy in spheroids treated with identical drug concentrations is reproducible. The use of Matrigel, which is easily accessible by pipette, enables the introduction of cells and other manipulations. Further investigation into establishing spheroid co‐culture systems is required to mimic cellular heterogeneity observed in tumour tissues. The limitations of our 3D culture system include the production of irregular, non‐circular spheroids of varying sizes and the inhomogeneity within individual wells.

Our study has several limitations. Although the combination of entinostat and tazemetostat demonstrated enhanced cytotoxicity and anti‐migratory effects in vitro, the lack of in vivo validation limits the translation of these findings to clinical settings. Additionally, this study utilised only MES‐SA and SK‐UT‐1 cell lines, and the results may not be broadly applicable to all subtypes of uterine sarcoma or patient‐derived tumour cells. Finally, further research is needed to establish 3D co‐culture systems that more accurately mimic the cellular heterogeneity of tumour tissues.

**FIGURE 5:**
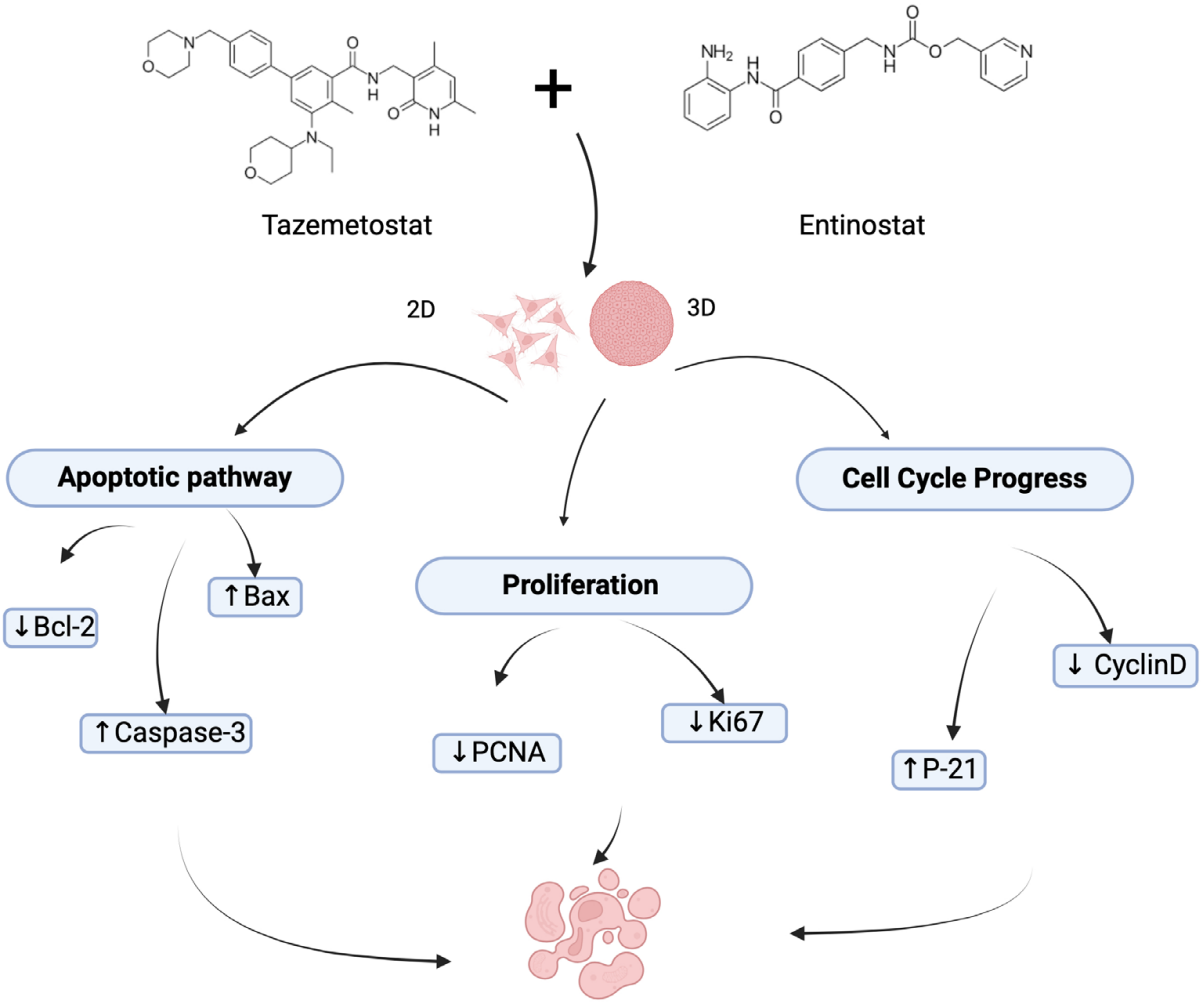
illustration of the study finding summary.

In conclusion, our studies demonstrate that entinostat and tazemetostat treatment alone showed a potent anti‐uterine sarcoma effect in both 2D and 3D culture conditions. Importantly, the combination of entinostat and tazemetostat is significantly superior to a single‐agent treatment. Data from this study suggest that dual targeting EZH2 and HDACs may provide a promising treatment option for this aggressive cancer (Figure 5).

## Author Contributions

**Mervat M. Omran:** data curation (lead), formal analysis (lead), investigation (lead), methodology (lead), resources (lead), software (lead), writing – original draft (lead), writing – review and editing (equal). **Somayeh Vafaei:** data curation (supporting), investigation (supporting), resources (supporting). **Samar Alkhrait:** writing – original draft (supporting), writing – review and editing (equal). **Qiwei Yang:** conceptualization (equal), funding acquisition (equal), project administration (equal), supervision (equal), visualization (equal), writing – review and editing (equal). **Ayman Al‐Hendy:** conceptualization (equal), funding acquisition (equal), project administration (equal), supervision (equal), writing – review and editing (equal).

## Ethics Statement

The authors have nothing to report.

## Consent

The manuscript was reviewed and approved by all the authors.

## Conflicts of Interest

The authors declare no conflicts of interest. Ayman Al‐Hendy reports support from the National Institutes of Health and has consulting and advisory relationships with Myovant Sciences Ltd. and Pfizer. Dr. Al‐Hendy is also the founder of the INOFFA Company.

## Supporting information


**Figure S1.** Time‐lapse imaging of uterine sarcoma spheroid formation.
**Figure S2.** Cytotoxicity analysis of HDAC and EZH2 inhibitors in MES‐SA uterine sarcoma cells. (A–D) Cytotoxicity analysis of vorinostat (0–100 μM) (A), tucidinostat (0–100 μM) (B), entinostat (0–100 μM) (C) and tazemetostat (0–200 μM) (D) in MES‐SA 2D cell culture after 24, 48 and 72 h treatments. (E–G) Surviving fraction of MES‐SA cells treated with a range of tazemetostat concentration (0–100 μM) combined with doses of 1.5, 3.1, 6.25 and 12.5 μM of vorinostat (E), tucidinostat (F), entinostat (G) using the MTT assay. Values are the mean ± SD of three independent experiments performed in triplicate. Statistical significance of results was analysed using one‐way ANOVA followed by Tukey’s multiple comparison test. *p < 0.05.
**Figure S3.** Cytotoxicity effects of (A) tazemetostat (0–100 μM), (B) entinostat (0–100 μM) on SK‐UT‐1 cell line after 24, 48 and 72 h treatments using MTT assay. Data represent mean ± SD from three independent experiments performed in triplicate. Statistical analysis was conducted using two‐way ANOVA followed by multiple comparison test. *p < 0.05.
**Figure S4.** Effect of tazemetostat 4.5 μM, entinostat 6.5 μM and their combination on mRNA expression of PITX2 (A, B) and CD40 (C, D) on both 2D and 3D culture systems on MES‐SA cells. Values are the mean ± SD of two independent experiments performed in duplicate. Statistical significance of results was analysed using one‐way ANOVA followed by Tukey’s multiple comparison test. *p < 0.05, **p < 0.01, ***p < 0.001. ‘C’ stands for control, ‘Taz’ for tazemetostat and ‘E’ for entinostat.

## Data Availability

The raw data were generated at the University of Chicago. Derived data supporting the findings of this study are available from the corresponding authors upon request.

## References

[1] S. E. Brooks , M. Zhan , T. Cote , and C. R. Baquet , “Surveillance, Epidemiology, and End Results Analysis of 2677 Cases of Uterine Sarcoma 1989‐1999,” Gynecologic Oncology 93, no. 1 (2004): 204–208, 10.1016/j.ygyno.2003.12.029. 15047237

[2] F. J. Major , J. A. Blessing , S. G. Silverberg , et al., “Prognostic Factors in Early‐Stage Uterine Sarcoma. A Gynecologic Oncology Group Study,” Cancer 71, no. 4 Suppl (1993): 1702–1709, 10.1002/cncr.2820710440. 8381710

[3] A. Rizzo , M. A. Pantaleo , M. Saponara , and M. Nannini , “Current Status of the Adjuvant Therapy in Uterine Sarcoma: A Literature Review,” World Journal of Clinical Cases 7, no. 14 (2019): 1753–1763, 10.12998/wjcc.v7.i14.1753. 31417921 PMC6692269

[4] C. D. Fletcher , “The Evolving Classification of Soft Tissue Tumours – An Update Based on the New 2013 WHO Classification,” Histopathology 64, no. 1 (2014): 2–11, 10.1111/his.12267. 24164390

[5] J. G. Zhou , H. T. Zhao , S. H. Jin , X. Tian , and H. Ma , “Identification of a RNA‐Seq‐Based Signature to Improve Prognostics for Uterine Sarcoma,” Gynecologic Oncology 155, no. 3 (2019): 499–507, 10.1016/j.ygyno.2019.08.033. 31662204

[6] Q. Yang , M. Ciebiera , M. V. Bariani , et al., “Comprehensive Review of Uterine Fibroids: Developmental Origin, Pathogenesis, and Treatment,” Endocrine Reviews 43 (2021): 678–719. 10.1210/endrev/bnab039 PMC9277653 34741454

[7] R. Margueron and D. Reinberg , “The Polycomb Complex PRC2 and Its Mark in Life,” Nature 469, no. 7330 (2011): 343–349. 21248841 10.1038/nature09784 PMC3760771

[8] A. Chase and N. C. Cross , “Aberrations of EZH2 in Cancer,” Clinical Cancer Research 17, no. 9 (2011): 2613–2618. 21367748 10.1158/1078-0432.CCR-10-2156

[9] M. A. Lunning and M. R. Green , “Mutation of Chromatin Modifiers; an Emerging Hallmark of Germinal Center B‐Cell Lymphomas,” Blood Cancer Journal 5 (2015): e361. 26473533 10.1038/bcj.2015.89 PMC4635197

[10] C. J. Sneeringer , M. P. Scott , K. W. Kuntz , et al., “Coordinated Activities of Wild‐Type Plus Mutant EZH2 Drive Tumor‐Associated Hypertrimethylation of Lysine 27 on Histone H3 (H3K27) in Human B‐Cell Lymphomas,” Proceedings of the National Academy of Sciences of the United States of America 107, no. 49 (2010): 20980–20985. 21078963 10.1073/pnas.1012525107 PMC3000297

[11] D. B. Yap , J. Chu , T. Berg , et al., “Somatic Mutations at EZH2 Y641 Act Dominantly Through a Mechanism of Selectively Altered PRC2 Catalytic Activity, to Increase H3K27 Trimethylation,” Blood 117, no. 8 (2011): 2451–2459. 21190999 10.1182/blood-2010-11-321208 PMC3062411

[12] S. Kailayangiri , B. Altvater , S. Lesch , et al., “EZH2 Inhibition in Ewing Sarcoma Upregulates GD2 Expression for Targeting With Gene‐Modified T Cells,” Molecular Therapy 27, no. 5 (2019): 933–946, 10.1016/j.ymthe.2019.02.014. 30879952 PMC6520468

[13] N. Zhang , Z. Zeng , S. Li , F. Wang , and P. Huang , “High Expression of EZH2 as a Marker for the Differential Diagnosis of Malignant and Benign Myogenic Tumors,” Scientific Reports 8 (2018): 12331, 10.1038/s41598-018-30648-7. 30120321 PMC6098067

[14] J. Huang , H. Gou , J. Yao , et al., “The Noncanonical Role of EZH2 in Cancer,” Cancer Science 112, no. 4 (2021): 1376–1382, 10.1111/cas.14840. 33615636 PMC8019201

[15] S. K. Knutson , S. Kawano , Y. Minoshima , et al., “Selective Inhibition of EZH2 by EPZ‐6438 Leads to Potent Antitumor Activity in EZH2‐Mutant Non‐Hodgkin Lymphoma,” Molecular Cancer Therapeutics 13, no. 4 (2014): 842–854. 24563539 10.1158/1535-7163.MCT-13-0773

[16] S. K. Knutson , N. M. Warholic , T. J. Wigle , et al., “Durable Tumor Regression in Genetically Altered Malignant Rhabdoid Tumors by Inhibition of Methyltransferase EZH2,” Proceedings of the National Academy of Sciences of the United States of America 110, no. 19 (2013): 7922–7927. 23620515 10.1073/pnas.1303800110 PMC3651445

[17] A. Italiano , J. C. Soria , M. Toulmonde , et al., “Tazemetostat, an EZH2 Inhibitor, in Relapsed or Refractory B‐Cell Non‐Hodgkin Lymphoma and Advanced Solid Tumours: A First‐In‐Human, Open‐Label, Phase 1 Study,” Lancet Oncology 19, no. 5 (2018): 649–659, 10.1016/S1470-2045(18)30145-1. 29650362

[18] M. Gounder , P. Schöffski , R. L. Jones , et al., “Tazemetostat in Advanced Epithelioid Sarcoma With Loss of INI1/SMARCB1: An International, Open‐Label, Phase 2 Basket Study,” Lancet Oncology 21, no. 11 (2020): 1423–1432, 10.1016/S1470-2045(20)30451-4. 33035459

[19] R. Straining and W. Eighmy , “Tazemetostat: EZH2 Inhibitor,” Journal of the Advanced Practitioner in Oncology 13, no. 2 (2022): 158–163, 10.6004/jadpro.2022.13.2.7. 35369397 PMC8955562

[20] Q. Yang , A. Falahati , A. Khosh , et al., “Targeting Class I Histone Deacetylases in Human Uterine Leiomyosarcoma,” Cells 11, no. 23 (2022): 3801, 10.3390/cells11233801. 36497061 PMC9735512

[21] F. Tang , E. Choy , C. Tu , F. Hornicek , and Z. Duan , “Therapeutic Applications of Histone Deacetylase Inhibitors in Sarcoma,” Cancer Treatment Reviews 59 (2017): 33–45, 10.1016/j.ctrv.2017.06.006. 28732326 PMC5581728

[22] A. J. de Ruijter , A. H. van Gennip , H. N. Caron , S. Kemp , and A. B. van Kuilenburg , “Histone Deacetylases (HDACs): Characterization of the Classical HDAC Family,” Biochemical Journal 370 (2003): 737–749. 12429021 10.1042/BJ20021321 PMC1223209

[23] B. Xu , Q. Zhang , X. Hu , et al., “Entinostat, a Class I Selective Histone Deacetylase Inhibitor, Plus Exemestane for Chinese Patients With Hormone Receptor‐Positive Advanced Breast Cancer: A Multicenter, Randomized, Double‐Blind, Placebo‐Controlled, Phase 3 Trial,” Acta Pharmaceutica Sinica B 13, no. 5 (2023): 2250–2258, 10.1016/j.apsb.2023.02.001. 37250148 PMC10213795

[24] N. Masuda , K. Tamura , H. Yasojima , et al., “Phase 1 Trial of Entinostat as Monotherapy and Combined With Exemestane in Japanese Patients With Hormone Receptor‐Positive Advanced Breast Cancer,” BMC Cancer 21, no. 1 (2021): 1269, 10.1186/s12885-021-08973-4. 34819039 PMC8611843

[25] R. M. Connolly , M. A. Rudek , and R. Piekarz , “Entinostat: A Promising Treatment Option for Patients With Advanced Breast Cancer,” Future Oncology 13, no. 13 (2017): 1137–1148, 10.2217/fon-2016-0526. 28326839 PMC5618943

[26] N. Ngamphaiboon , G. K. Dy , W. W. Ma , et al., “A Phase I Study of the Histone Deacetylase (HDAC) Inhibitor Entinostat, in Combination With Sorafenib in Patients With Advanced Solid Tumors,” Investigational New Drugs 33, no. 1 (2015): 225–232, 10.1007/s10637-014-0174-6. 25371323

[27] M. Kapałczyńska , T. Kolenda , W. Przybyła , et al., “2D and 3D Cell Cultures – A Comparison of Different Types of Cancer Cell Cultures,” Archives of Medical Science 14, no. 4 (2018): 910–919, 10.5114/aoms.2016.63743. 30002710 PMC6040128

[28] J. A. Mather , “Ethics and Care: For Animals, Not Just Mammals,” Animals 9 (2019): 1018, 10.3390/ani9121018. 31766726 PMC6941085

[29] S. Banerjee , W. Xu , I. Chowdhury , et al., “Human Myometrial and Uterine Fibroid Stem Cell‐Derived Organoids for Intervening the Pathophysiology of Uterine Fibroid,” Reproductive Sciences 29, no. 9 (2022): 2607–2619. 35585291 10.1007/s43032-022-00960-9 PMC9444830

[30] M. Ali , D. Stone , A. Laknaur , Q. Yang , and A. Al‐Hendy , “EZH2 Activates Wnt/β‐Catenin Signaling in Human Uterine Fibroids, Which Is Inhibited by the Natural Compound Methyl Jasmonate,” F&S Science 4, no. 3 (2023): 239–256, 10.1016/j.xfss.2023.05.003. 37182601 PMC10527015

[31] W. J. Magner , A. L. Kazim , C. Stewart , et al., “Activation of MHC Class I, II, and CD40 Gene Expression by Histone Deacetylase Inhibitors,” Journal of Immunology 165, no. 12 (2000): 7017–7024, 10.4049/jimmunol.165.12.7017. 11120829

[32] C. J. Gregorie , J. L. Wiesen , W. J. Magner , A. W. Lin , and T. B. Tomasi , “Restoration of Immune Response Gene Induction in Trophoblast Tumor Cells Associated With Cellular Senescence,” Journal of Reproductive Immunology 81, no. 1 (2009): 25–33, 10.1016/j.jri.2009.02.009. 19493573 PMC2731570

[33] L. Gore , M. L. Rothenberg , C. L. O'Bryant , et al., “A Phase I and Pharmacokinetic Study of the Oral Histone Deacetylase Inhibitor, MS‐275, in Patients With Refractory Solid Tumors and Lymphomas,” Clinical Cancer Research 14, no. 14 (2008): 4517–4525. 18579665 10.1158/1078-0432.CCR-07-1461 PMC2813676

[34] H. Hess‐Stumpp , T. U. Bracker , D. Henderson , and O. Politz , “MS‐275, a Potent Orally Available Inhibitor of Histone Deacetylases—The Development of an Anticancer Agent,” International Journal of Biochemistry & Cell Biology 39, no. 7–8 (2007): 1388–1405. 17383217 10.1016/j.biocel.2007.02.009

[35] G. J. Sabnis , O. G. Goloubeva , A. A. Kazi , P. Shah , and A. H. Brodie , “HDAC Inhibitor Entinostat Restores Responsiveness of Letrozole‐Resistant MCF‐7Ca Xenografts to Aromatase Inhibitors Through Modulation of Her‐2,” Molecular Cancer Therapeutics 12, no. 12 (2013): 2804–2816. 24092810 10.1158/1535-7163.MCT-13-0345 PMC3858401

[36] J. Lee , C. Bartholomeusz , O. Mansour , et al., “A Class I Histone Deacetylase Inhibitor, Entinostat, Enhances Lapatinib Efficacy in HER2‐Overexpressing Breast Cancer Cells Through FOXO3‐Mediated Bim1 Expression,” Breast Cancer Research and Treatment 146, no. 2 (2014): 259–272. 24916181 10.1007/s10549-014-3014-7 PMC4119423

[37] D. A. Yardley , R. R. Ismail‐Khan , B. Melichar , et al., “Randomized Phase II, Double‐Blind, Placebo‐Controlled Study of Exemestane With or Without Entinostat in Postmenopausal Women With Locally Recurrent or Metastatic Estrogen Receptor‐Positive Breast Cancer Progressing on Treatment With a Nonsteroidal Aromatase Inhibitor,” Journal of Clinical Oncology 31, no. 17 (2013): 2128–2135. 23650416 10.1200/JCO.2012.43.7251 PMC4881332

[38] R. T. Kurmasheva , A. Bandyopadhyay , E. Favours , et al., “Evaluation of Entinostat Alone and in Combination With Standard‐of‐Care Cytotoxic Agents Against Rhabdomyosarcoma Xenograft Models,” Pediatric Blood & Cancer 66, no. 8 (2019): e27820, 10.1002/pbc.27820. 31099166 PMC6685061

[39] J. K. Jamison , M. Zhou , E. P. Gelmann , et al., “Entinostat in Patients With Relapsed or Refractory Abdominal Neuroendocrine Tumors,” Oncologist 29 (2024): oyae118, 10.1093/oncolo/oyae118. PMC11379646 38886159

[40] M. M. Omran , S. Vafaei , A. Al‐Hendy , and Q. Yang , “Combined Inhibition of EZH2 and Histone Deacetylases as a Potential Epigenetic Therapy for Human Uterine Sarcoma Cells,” Journal of Clinical Oncology 41 (2023): e17620, 10.1200/JCO.2023.41.16_suppl.e17620.

[41] Y. Ma , M. Baltezor , L. Rajewski , et al., “Targeted Inhibition of Histone Deacetylase Leads to Suppression of Ewing Sarcoma Tumor Growth Through an Unappreciated EWS‐FLI1/HDAC3/HSP90 Signaling Axis,” Journal of Molecular Medicine (Berlin, Germany) 97, no. 7 (2019): 957–972, 10.1007/s00109-019-01782-0. 31025088 PMC6584050

[42] A. Solta , K. Boettiger , I. Kovács , et al., “Entinostat Enhances the Efficacy of Chemotherapy in Small Cell Lung Cancer Through S‐Phase Arrest and Decreased Base Excision Repair,” Clinical Cancer Research 29, no. 22 (2023): 4644–4659, 10.1158/1078-0432.CCR-23-1795. 37725585 PMC10644001

[43] S. Sultana , O. Uehara , K. Yoshida , T. Saito , and Y. Abiko , “The Histone Deacetylase Inhibitor, Entinostat (MS‐275), Induces the Odontogenic Differentiation of an Odontoblast‐Like Cell Line in the Absence of an Osteoblast Mineralization Medium,” Odontology 109, no. 3 (2021): 661–671, 10.1007/s10266-020-00588-8. 33475895

[44] Epizyme Inc ., “Tazverik (Tazemetostat) Package Insert,” (2020), https://www.tazverik.com/Content/pdf/prescribing‐information.pdf.

[45] FDA , accessed August 20, 2024, https://www.fda.gov/news‐events/press‐announcements/fda‐approves‐first‐treatment‐option‐specifically‐patients‐epithelioid‐sarcoma‐rare‐soft‐tissue#:~:text=Tazverik%20was%20granted%20Accelerated%20Approval,of%20Tazverik%20to%20Epizyme%20Inc.

[46] D. L. Kolin , F. Dong , M. Baltay , et al., “SMARCA4‐Deficient Undifferentiated Uterine Sarcoma (Malignant Rhabdoid Tumor of the Uterus): A Clinicopathologic Entity Distinct From Undifferentiated Carcinoma,” Modern Pathology 31 (2018): 1442–1456. 29700418 10.1038/s41379-018-0049-z

[47] S. Bose , G. K. Schwartz , and M. Ingham , “Novel Therapeutics in the Treatment of Uterine Sarcoma,” American Society of Clinical Oncology Educational Book 42 (2022): 900–909, 10.1200/EDBK_350541. 35714303

[48] E. Julia and G. Salles , “EZH2 Inhibition by Tazemetostat: Mechanisms of Action, Safety and Efficacy in Relapsed/Refractory Follicular Lymphoma,” Future Oncology 17, no. 17 (2021): 2127–2140, 10.2217/fon-2020-1244. 33709777 PMC9892962

[49] X. Tan , Z. Zhang , P. Liu , H. Yao , L. Shen , and J. S. Tong , “RETRACTED ARTICLE: Inhibition of EZH2 Enhances the Therapeutic Effect of 5‐FU via PUMA Upregulation in Colorectal Cancer,” Cell Death & Disease 11 (2020): 1061, 10.1038/s41419-020-03266-3. 33311453 PMC7733595

[50] S. H. Hong , H. J. Hwang , D. H. Son , E. S. Kim , S. Y. Park , and Y. E. Yoon , “Inhibition of EZH2 Exerts Antitumorigenic Effects in Renal Cell Carcinoma via LATS1,” FEBS Open Bio 13, no. 4 (2023): 724–735, 10.1002/2211-5463.13579. PMC10068324 36808829

[51] A. A. Chomiak , R. L. Tiedemann , Y. Liu , et al., “Select EZH2 Inhibitors Enhance Viral Mimicry Effects of DNMT Inhibition Through a Mechanism Involving NFAT:AP‐1 Signaling,” Science Advances 10 (2024): eadk4423, 10.1126/sciadv.adk4423. 38536911 PMC10971413

[52] F. X. Xu , R. Sun , R. Owens , K. Hu , and D. Fu , “Assessing Drug Uptake and Response Differences in 2D and 3D Cellular Environments Using Stimulated Raman Scattering Microscopy,” preprint, bioRxiv, April 26, 2024, 10.1101/2024.04.22.590622. PMC12358159 39186736

[53] C. Berrouet , N. Dorilas , K. A. Rejniak , and N. Tuncer , “Comparison of Drug Inhibitory Effects (IC_50_) in Monolayer and Spheroid Cultures,” Bulletin of Mathematical Biology 82, no. 6 (2020): 68, 10.1007/s11538-020-00746-7. 32495209 PMC9773863

[54] K. Duval , H. Grover , L. H. Han , et al., “Modeling Physiological Events in 2D vs. 3D Cell Culture,” Physiology (Bethesda) 32, no. 4 (2017): 266–277, 10.1152/physiol.00036.201. 28615311 PMC5545611

