# Supplementary material for: Dual Targeting EZH2 and Histone Deacetylases in Human Uterine Sarcoma Cells Under Both 2D and 3D Culture Conditions
Source: J Cell Mol Med. 2025 Jun 4;29(11):e70626. doi: 10.1111/jcmm.70626 (PMC12135698; doi:10.1111/jcmm.70626)
Supplement: Supplementary file 1 — Figure S1. Time‐lapse imaging of uterine sarcoma spheroid formation. Figure S2. Cytotoxicity analysis of HDAC and EZH2 inhibitors in MES‐SA uterine sarcoma cells. (A–D) Cytotoxicity analysis of vorinostat (0–100 μM) (A), tucidinostat (0–100 μM) (B), entinostat (0–100 μM) (C) and tazemetostat (0–200 μM) (D) in MES‐SA 2D cell culture after 24, 48 and 72 h treatments. (E–G) Surviving fraction of MES‐SA cells treated with a range of tazemetostat concentration (0–100 μM) combined with doses of 1.5, 3.1, 6.25 and 12.5 μM of vorinostat (E), tucidinostat (F), entinostat (G) using the MTT assay. Values are the mean ± SD of three independent experiments performed in triplicate. Statistical significance of results was analysed using one‐way ANOVA followed by Tukey’s multiple comparison test. *p < 0.05. Figure S3. Cytotoxicity effects of (A) tazemetostat (0–100 μM), (B) entinostat (0–100 μM) on SK‐UT‐1 cell line after 24, 48 and 72 h treatments using MTT assay. Data represent mean ± SD from three independent experiments performed in triplicate. Statistical analysis was conducted using two‐way ANOVA followed by multiple comparison test. *p < 0.05. Figure S4. Effect of tazemetostat 4.5 μM, entinostat 6.5 μM and their combination on mRNA expression of PITX2 (A, B) and CD40 (C, D) on both 2D and 3D culture systems on MES‐SA cells. Values are the mean ± SD of two independent experiments performed in duplicate. Statistical significance of results was analysed using one‐way ANOVA followed by Tukey’s multiple comparison test. *p < 0.05, **p < 0.01, ***p < 0.001. ‘C’ stands for control, ‘Taz’ for tazemetostat and ‘E’ for entinostat. [file JCMM-29-e70626-s001.docx]

Supplementary Figure (Supplementary Figure 1):


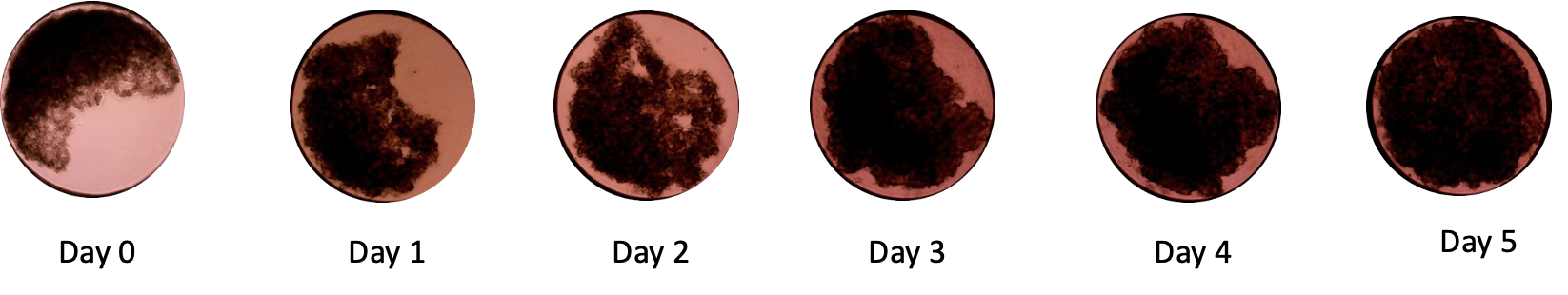


**Fig S1:(Caption of the Figure)** Time lapse imaging of Uterine sarcoma spheroid formation.

Supplementary Figure (Supplementary Figure 2):

**Fig S2: (Caption of the Figure)** Cytotoxicity effects of Vorinostat (0-100µM)(A), Tucidinostat(0-100µM) (B), Entinostat (0-100µM) (C) and Tazemetostat(0-200µM) (D) in MES-SA 2D cell culture after 24, 48, 72 hour treatments. Surviving fraction of MES-SA cells treated with range of concentration (0-100µM) Tazemetostat combined with 1.5, 3.1, 6.25, 12.5 µM of Vorinostat (E), Tucidinostat (F), Entinostat (G) using MTT assay. Data represent mean ± SD from three independent experiments performed in triplicate. Statistical analysis was conducted using one-way ANOVA followed by Tukey’s multiple comparison test. **p* < 0.05

Supplementary Figure (Supplementary Figure 3):

**Fig S3:** (Caption of the Figure) Cytotoxicity effects of (A) Tazemetostat(0-100µM)(B) Entinostat (0-100µM) on SK-UT-1 cell line after 24, 48, 72 hour Treatments using MTT assay. Data represent mean ± SD from three independent experiments performed in triplicate. Statistical analysis was conducted using Two-way ANOVA followed by multiple comparison test. *p < 0.05

Supplementary Figure (Supplementary Figure 4):

**Fig S4:** Effect of Tazemetostat , Entinostat, and their combination on mRNA expression of *PITX2 and CD40* in both 2D and 3D culture systems. mRNA expression of *PITX2* (A, B) and *CD40* (C, D) in MES-SA cells cultured in 2D and 3D systems following treatment with Tazemetostat (4.5 µM), Entinostat (6.5 µM), or their combination. Data represent means±SD from two independent experiments performed in duplicate. Statistical anakysis was perfromed using one-way ANOVA followed by Tukey’s multiple comparison test. *p < 0.05,**p < 0.01, ***p < 0.001. “C” stands for control; “Taz” for Tazemetostat; “E” for Entinostat.
